# 

**Published:** 1884-08

**Authors:** 


### CONTENTS:

ART. I.—The Teeth and Their Treatment. By Elton R. Smilie. M. D. ............ 145
ART. II—Micro-Organisms and Dental Caries. By Dr. W. D. Miller. ............ 164
ART. III—Repairing Vulcanite Plates. By Geo. B. Snow, D. D. S. ............ 173
Communication from Dr. D. Genese ............ 180

### EDITORIAL, ETC.

Stomatoscopes ............ 182

### MONTHLY SUMMARY.

Cleanliness ............ 185

Large Salivary Calculus ............ 187

Cast Metal Plates ............ 188

Are the Anterior Columns Excitable ............ 189

Tooth Brushes—What They Should Be, Etc ............ 189

Micrococci Causing Loss of Hair ............ 190

Mechanical Skill Necessary to Dentists ............ 190

A Dangerous Adulteration of Iodoform ............ 191

The Care of Teeth ............ 191

Loose Teeth on Plates ............ 192

